# Peer-mediated instruction improves acquisition of complex wrestling techniques in school physical education

**DOI:** 10.1371/journal.pone.0348070

**Published:** 2026-05-22

**Authors:** Salih Gürsoy, Aylin Zekioglu

**Affiliations:** 1 Physical Education Teacher, Ministry of National Education, Manisa, Turkey; 2 Faculty of Sport Sciences, Manisa Celal Bayar University, Manisa, Turkey; Balikesir University: Balikesir Universitesi, TÜRKIYE

## Abstract

This study investigated the effectiveness of peer-mediated instruction as a student-centered teaching method in physical education by assessing its impact on middle school students’ ability to learn complex wrestling techniques. A total of 36 students took part in an eight-week program comparing peer-mediated instruction with traditional direct instruction. Performance on four wrestling techniques—arm drag, underarm pass, single-leg takedown, and double-leg takedown—was evaluated using standardized observation criteria and independent raters. Results from independent-samples t-tests indicated that students in the peer-teaching group performed significantly better on the underarm pass, t(34) = 3.976, p = .001, d = 1.33, and the double-leg takedown, t(34) = 2.31, p = .027, d = 0.78. No significant differences between groups were found for the arm drag, t(34) = 1.685, p = .101, d = 0.55, or the single-leg takedown, t(34) = 0.52, p = .606, d = 0.18. These results suggest that peer-mediated instruction may be more effective than direct instruction for techniques that demand higher perceptual-motor coordination and involve greater task complexity. The study adds to the sport pedagogy literature by demonstrating that peer-assisted learning can support skill development and collaborative participation in school-based physical education.

## Introduction

Wrestling holds an important place in both traditional and modern Turkish sport culture and requires a combination of technical precision, coordination, tactical awareness, and psychomotor skills [1,2]. In school-based physical education, the choice of instructional approach plays a critical role in students’ motor skill development and learning retention [3,4]. While teacher-centered direct instruction remains widely used, there has been increasing emphasis on student-centered approaches that promote active participation and shared responsibility for learning [5,6].

Among these approaches, peer-mediated instruction has demonstrated positive effects on skill acquisition and engagement across various physical education contexts [7,8], although its application in wrestling remains relatively underexplored.

The peer-teaching model involves learners acting as both instructors and students, promoting social interaction, cooperative problem-solving, and reciprocal feedback [9,10]. This approach aligns with principles of motor learning, where skill development is strengthened through repetition, modeling, and performance feedback [11]. Additionally, self-determination theory suggests that peer-mediated learning can enhance intrinsic motivation by supporting learners’ autonomy, competence, and relatedness [12].

However, in combat sports such as wrestling, techniques involve complex body positioning, close physical contact, and sequential movement patterns that require advanced coordination and timing [1]. Previous research on motor skill acquisition also suggests that tasks requiring higher levels of perceptual–motor coordination and decision-making impose greater cognitive and physical demands on learners [11,13]. These characteristics indicate that instructional effectiveness may vary depending on the complexity of the skill and the cognitive demands involved.

In addition to its instructional value, peer teaching can be considered within the broader framework of sustainable education, which emphasizes collaborative learning, shared responsibility, and learner autonomy [14,15]. In physical education settings, such approaches may contribute to more inclusive and participatory learning environments. However, rather than positioning peer teaching as a superior method, the present study considers it as a complementary instructional strategy that may support engagement and skill development under specific conditions.

The purpose of this study is to evaluate the effectiveness of peer teaching versus direct instruction in teaching four key wrestling techniques (arm drag, underarm pass, single-leg takedown, double-leg takedown) to middle school students. The main research question is:

Is peer teaching more effective than direct instruction in helping students learn wrestling techniques, and does its effectiveness vary depending on the type of skill?

It was hypothesized that peer-mediated instruction would lead to higher technical performance than direct instruction, with a stronger effect for techniques requiring greater coordination and complexity.

This study seeks to enhance sport pedagogy and wrestling education by providing empirical evidence for applying student-centered instructional models in a combat sport setting. Additionally, this research advocates sustainability in education by viewing peer teaching not just as a teaching method but as a resilient learning framework that shares responsibility, fosters collaboration, and strengthens long-term educational sustainability.

Sport pedagogy research increasingly emphasizes instructional models that promote active learner engagement and shared responsibility for learning. Within this context, peer-mediated instructional approaches have gained attention as strategies that support both cognitive and motor learning processes in physical education. By allowing students to collaboratively observe, explain, and evaluate movement patterns, peer teaching may enhance perceptual-motor processing and reinforce skill acquisition through repeated feedback cycles. Despite these theoretical advantages, empirical evidence examining the effectiveness of peer teaching for learning complex combat sport skills remains limited.

## Materials and methods

### Research design

A posttest-only quasi-experimental design was used to compare the effects of the peer-teaching and direct-instruction models on students’ learning of fundamental wrestling techniques. Two instructional conditions were implemented, with direct instruction serving as the comparison group rather than a traditional control group. The instructional intervention lasted eight weeks and was conducted twice per week, with each session lasting approximately 40 minutes. The study was conducted between 12/01/2022 and 09/03/2022.

### Participants

The sample included 36 middle school students (ages 12–14; 50% female) in grades 6–8 who had never tried wrestling before. Participants were recruited through convenience sampling at a public school. Written informed consent was obtained from both students and their parents before participation. Inclusion criteria required that participants had no prior formal wrestling training and were actively enrolled in physical education classes. Students with prior wrestling experience or any medical condition preventing physical activity were excluded from the study.

The sample size was determined based on accessibility within the school context and the voluntary participation of students. Although no a priori power analysis was conducted, the sample size was considered sufficient to detect medium to large effects, consistent with similar studies in physical education research.

### Instructional ıntervention

Both groups learned the same four techniques: arm drag, underarm pass, single-leg takedown, and double-leg takedown. The direct instruction group was taught by the physical education teacher through demonstration and corrective feedback, while the peer teaching group received instruction from trained peer tutors who followed structured peer-interaction protocols. To ensure implementation fidelity, all sessions were video-recorded and reviewed by subject-matter experts.

Each instructional session followed a structured format consisting of demonstration, guided practice, and feedback phases. In the peer-teaching group, peer tutors were assigned specific instructional roles, including demonstrating techniques, providing corrective feedback, and guiding practice using structured task cues. Peer tutors received prior orientation on how to deliver feedback and monitor performance. In contrast, the direct instruction group followed a teacher-centered approach where the teacher provided demonstrations, instructions, and corrective feedback throughout the sessions. All sessions were standardized in terms of duration and content to ensure consistency across groups.

### Ethical considerations

Ethical approval for the study was initially obtained from the Ethics Committee of Manisa Celal Bayar University on **11/03/2020** (Approval No: **11/03/2020/20.478.486**). Due to a change in the study location, the same committee issued an additional ethics decision on **12/01/2022** (Decision No: **1160**). Written informed consent was obtained from all participants and their parents prior to participation. Participant confidentiality and voluntary participation were ensured throughout the study.

### Measurement

Performance was assessed using the Wrestling Observation Form, developed with input from three academic experts and three experienced wrestling coaches. The instrument included 23 items rated on a 3-point scale (1 = unsuccessful, 2 = partially successful, 3 = successful). Content validity was verified by expert review, and pilot testing with 78 young wrestlers demonstrated high internal consistency for the technique-based subscales (Cronbach’s alpha: 0.764–0.940).

Performance evaluations were conducted by independent raters who were blinded to group assignments and were not involved in the instructional process. All performances were assessed using anonymized participant codes. Inter-rater scoring procedures were standardized before evaluation sessions to ensure consistent application of the observation rubric, thereby improving objectivity in the assessment of technical performance.

The development and use of structured observation forms for performance assessment are widely supported in physical education and sport science research [16,17].

### Data analysis

All statistical analyses were conducted using SPSS (Version 25.0, IBM Corp., Armonk, NY, USA). Assumptions of normality were assessed using Shapiro–Wilk tests and skewness and kurtosis values. Independent samples t-tests compared post-test scores between groups for each technique. Effect sizes (ES) were calculated using Cohen’s d, where values of 0.2, 0.5, and 0.8 indicate small, medium, and large effects, respectively. The significance level was set at p < .05 (Table 1)‌‌.

**Table 1:** Participant characteristics.

Variable	n	%
Total Participants	36	100
Female	18	50
Male	18	50
Age (years) → 13.1 ± 0.8	36	100

*Note.* All students were middle school students with no prior wrestling experience.

## Results

As shown in Table 2, the internal consistency coefficients for all techniques ranged between.764 and.940, indicating acceptable to high reliability. These findings suggest that the performance evaluation tool produced consistent scores across technique-specific assessments. In turn, this strengthens confidence in the group comparisons by reducing the likelihood that the observed differences were influenced by measurement error rather than actual performance differences.

**Table 2:** Internal reliability of performance ratings.

Technique	Cronbach’s α	Interpretation
Arm Drag	.829	Good
Underarm Pass	.764	Acceptable
Single-Leg Takedown	.838	Good
Double-Leg Takedown	.827	Good
Overall	.940	Excellent

*Note.* Reliability values above.70 indicate acceptable internal consistency.

As shown in Table 3, students in the peer-teaching group demonstrated higher mean performance scores than those in the direct instruction group across all techniques, with the largest differences observed in the underarm pass and double-leg takedown. These descriptive differences further support the pattern observed in the inferential analysis. Confidence intervals were not included in the current analysis; however, effect size estimates were reported to support the interpretation of the findings.

**Table 3:** Descriptive statistics of performance scores.

Skill	Peer Teaching M (SD)	Direct Instruction M (SD)
Arm Drag	1.41 (.36)	1.21 (.34)
Underarm Pass	1.41 (.24)	1.01 (.34)
Single-Leg Takedown	1.28 (.26)	1.22 (.37)
Double-Leg Takedown	1.42 (.24)	1.15 (.42)

*Note.* Scores ranged from 1 to 3, with higher scores indicating better performance.

Table 4 presents the results of independent-samples t-tests, indicating statistically significant differences between groups for the underarm pass and the double-leg takedown. In contrast, no significant differences were found for the arm drag and single-leg takedown.

**Table 4:** Independent samples *t* test results.

Skill	t(34)	p	Cohen’s d	Effect size
Underarm Pass	3.976	.001	1.33	Large
Double-Leg Takedown	2.31	.027	0.78	Medium
Arm Drag	1.685	.101	0.55	Medium
Single-Leg Takedown	0.52	.606	0.18	Small

*Note.* Cohen’s d values were interpreted as small (0.2), medium (0.5), and large (0.8).

The largest effect size was observed in the underarm pass (d = 1.33), indicating a substantial practical difference between instructional methods. The double-leg takedown also demonstrated a meaningful effect (d = 0.78), suggesting moderate practical relevance. In contrast, the smallest effect size was found in the single-leg takedown (d = 0.18), indicating minimal practical difference between the instructional approaches. Although the arm drag did not reach statistical significance, the effect size (d = 0.55) suggests a moderate practical difference that may warrant consideration in applied settings.

## Discussion

The aim of this study was to examine the effectiveness of peer-mediated instruction compared to direct instruction in teaching fundamental wrestling techniques. The findings indicated that peer teaching was more effective for techniques requiring higher levels of coordination and complexity, such as the underarm pass and double-leg takedown. In contrast, no significant differences were observed for simpler techniques, including the arm drag and single-leg takedown. These results suggest that the effectiveness of instructional approaches may vary depending on the motor and cognitive demands of the skill.

These findings are partially consistent with previous research demonstrating that peer-mediated instruction enhances motor skill acquisition through increased engagement and feedback opportunities [8,9,18]. However, unlike studies reporting generalized improvements across tasks, the present findings suggest that the effectiveness of peer teaching may depend on task complexity. This discrepancy may be explained by the nature of the skills examined. In the current study, more complex techniques required higher levels of coordination, timing, and perceptual processing, which may have benefited more from interactive and feedback-rich learning environments. In contrast, simpler techniques may not require the same level of instructional interaction, allowing direct instruction to be equally effective.

From a motor learning perspective, complex skills benefit from instructional environments that encourage observation, feedback, and active participation. Peer teaching gives learners opportunities to observe peers performing the same task, exchange feedback in clear language, and collaborate to correct errors. These processes are known to support motor schema development and flexible movement control, especially for skills involving real-time decision-making and coordination [11]. Conversely, techniques characterized by more predictable movement patterns and lower cognitive demands can be effectively learned through demonstration and repetition alone, which may explain the similar outcomes seen with the arm drag and single-leg takedown.

The current findings support previous research in physical education showing that peer-mediated instructional models enhance skill development, engagement, and depth of learning, especially for tasks involving higher-level motor and cognitive skills [5,7,19]. By applying this evidence to wrestling—a contact sport with limited educational research—this study provides new insights into how student-centered teaching models can be implemented in contact sports.

Beyond immediate performance outcomes, peer teaching can be seen as a socially and pedagogically sustainable instructional approach. By sharing instructional responsibility among learners rather than relying only on teacher-led instruction, peer teaching encourages autonomy, collaboration, and shared ownership of learning. This approach builds resilient learning environments where students actively support each other’s development, boosting social cohesion and sustained engagement in physical education settings. These qualities align with broader sustainability frameworks in education that highlight participatory learning, collective responsibility, and the growth of transferable interpersonal skills [14,15]. From a sustainability perspective, peer teaching redistributes instructional responsibility within the learning environment, reducing reliance on a single instructional authority and promoting collaborative knowledge building. Such structures support socially sustainable learning environments where students actively participate in the learning process and develop transferable interpersonal skills. In physical education settings, this approach can help create resilient learning systems capable of maintaining engagement and skill growth even in contexts with limited instructional resources.

Overall, the findings indicate that peer teaching can be especially effective for learning complex wrestling techniques that require higher levels of coordination, perceptual awareness, and adaptive motor control. For educators, a hybrid teaching approach may be most effective, combining direct instruction for basic skills with peer-assisted learning for more advanced techniques. This balanced method enables teachers to improve learning conditions while promoting sustainable, student-centered practices in physical education programs. These results suggest that peer teaching can help create more sustainable learning environments in school-based physical education by encouraging shared instructional responsibility, collaborative engagement, and learner autonomy.

The findings of this study add to the growing body of research emphasizing the educational benefits of peer-mediated instruction in physical education. While teacher-centered teaching remains crucial for introducing basic movement patterns, peer-assisted learning settings can offer additional opportunities for observation, collaborative feedback, and repeated practice of tasks. These mechanisms are especially important for complex motor skills that require dynamic coordination and flexible movement control. Future research may further investigate how peer teaching interacts with task complexity, learner experience, and instructional design across various sports contexts.

In addition to supporting motor learning processes, the findings of this study can also be interpreted within the framework of observational learning and social interaction. Peer-mediated instruction allows learners to engage in active observation, verbalization, and correction of movement patterns, which are key mechanisms in skill acquisition. Previous research has shown that learning environments involving peer interaction can enhance both cognitive processing and motor performance by promoting error detection and immediate feedback [9,13].

Furthermore, the effectiveness of peer teaching observed in complex techniques may be explained by increased opportunities for distributed practice and task-specific feedback. In peer-assisted settings, learners are exposed to multiple demonstrations and corrective inputs, which may facilitate deeper encoding of movement patterns compared to traditional teacher-led instruction. This aligns with motor learning theories emphasizing variability of practice and augmented feedback as critical factors in skill acquisition [11].

Another important consideration is the role of social interaction in enhancing motivation and engagement. Peer-mediated environments can foster a sense of shared responsibility and collaborative learning, which may positively influence students’ willingness to participate and persist in challenging tasks. This is particularly relevant in school-based physical education, where affective and social factors play a significant role in learning outcomes. Therefore, the benefits of peer teaching may extend beyond motor performance to include motivational and social dimensions of learning. Taken together, these findings are consistent with recent evidence indicating that cooperative and peer-based instructional models in physical education contribute to improvements in not only motor performance but also social and cognitive learning outcomes [20].

### Limitations

This study has several limitations that should be considered when interpreting the findings. First, the relatively small sample size and the use of only one school setting may limit how broadly the results can be applied. Second, the study focused on short-term improvements in wrestling techniques rather than long-term retention of motor skills. Future research could involve larger and more diverse samples and explore the long-term educational and psychological effects of peer teaching in physical education settings.

## Conclusion‌‌

This study demonstrated that peer teaching can be an effective method in physical education, particularly for complex wrestling techniques that require higher levels of coordination, perceptual awareness, and adaptive motor control. Students who received peer-mediated instruction performed better on the underarm pass and double-leg takedown, while there were no significant differences for simpler techniques learned through direct instruction. These findings suggest that the effectiveness of teaching may depend on the interplay between the instructional method and the skill’s difficulty.

Beyond motor performance outcomes, the findings support peer teaching as a sustainable instructional method. By sharing responsibility among learners, peer teaching encourages active participation, shared ownership of learning, and collaborative problem-solving. Such student-centered environments may result in long-term educational resilience, sustained engagement, and the development of transferable interpersonal skills within physical education settings.

Overall, incorporating peer teaching into school-based physical education, especially when teaching complex motor skills, can improve learning outcomes and promote more sustainable instructional methods. A hybrid approach that combines direct instruction for basic skills with peer-mediated learning for more advanced techniques seems to provide a balanced and effective solution. These findings contribute to the growing literature in sport pedagogy by demonstrating that peer-mediated instructional strategies can effectively support the acquisition of complex motor skills in school-based physical education settings.

### Practical ımplications

Based on the findings of this study, several practical recommendations can be offered to physical education teachers, wrestling coaches, and curriculum developers.

- Peer teaching should be prioritized for complex techniques that require coordination, adaptive decision-making, and perceptual–motor integration (e.g., underarm pass, double-leg takedown).
- Direct instruction may remain sufficient for teaching basic or mechanically simple skills that rely primarily on repetition and demonstration.
- Peer tutors should undergo brief, structured training in communication, delivering feedback, and demonstrating movement to enhance their instructional effectiveness.
- Hybrid instructional models that combine teacher-led demonstrations with peer-mediated practice may provide optimal learning conditions in physical education.
- Encouraging students to take active teaching roles can enhance autonomy, motivation, and engagement while supporting sustainable, learner-centered educational environments.

From an educational policy perspective, incorporating peer-mediated instructional models into physical education curricula can promote more inclusive, participatory, and sustainable learning environments in schools.

Future research should examine the long-term retention of motor skills learned through peer teaching, its significance across different age groups and sports, and its broader psychological and social impacts in physical education settings.

## Supporting information

S1 File English-language dataset supporting the findings of this study. (XLSX)
